# Supplementary material for: DMRforPairs: identifying Differentially Methylated Regions between unique samples using array based methylation profiles
Source: BMC Bioinformatics. 2014 May 15;15:141. doi: 10.1186/1471-2105-15-141 (PMC4046028; doi:10.1186/1471-2105-15-141)
Supplement: Additional file 2 — DMRforPairs output for the comparison of A431-MCF7 and NA17018-NA17105. Please start from the HTML files in each folder. Available via the BMC Bioinformatics website. [file 1471-2105-15-141-S2.zip › 1394847754114233_MOESM2_ESM/A431_MCF7/figures/9822.pdf]

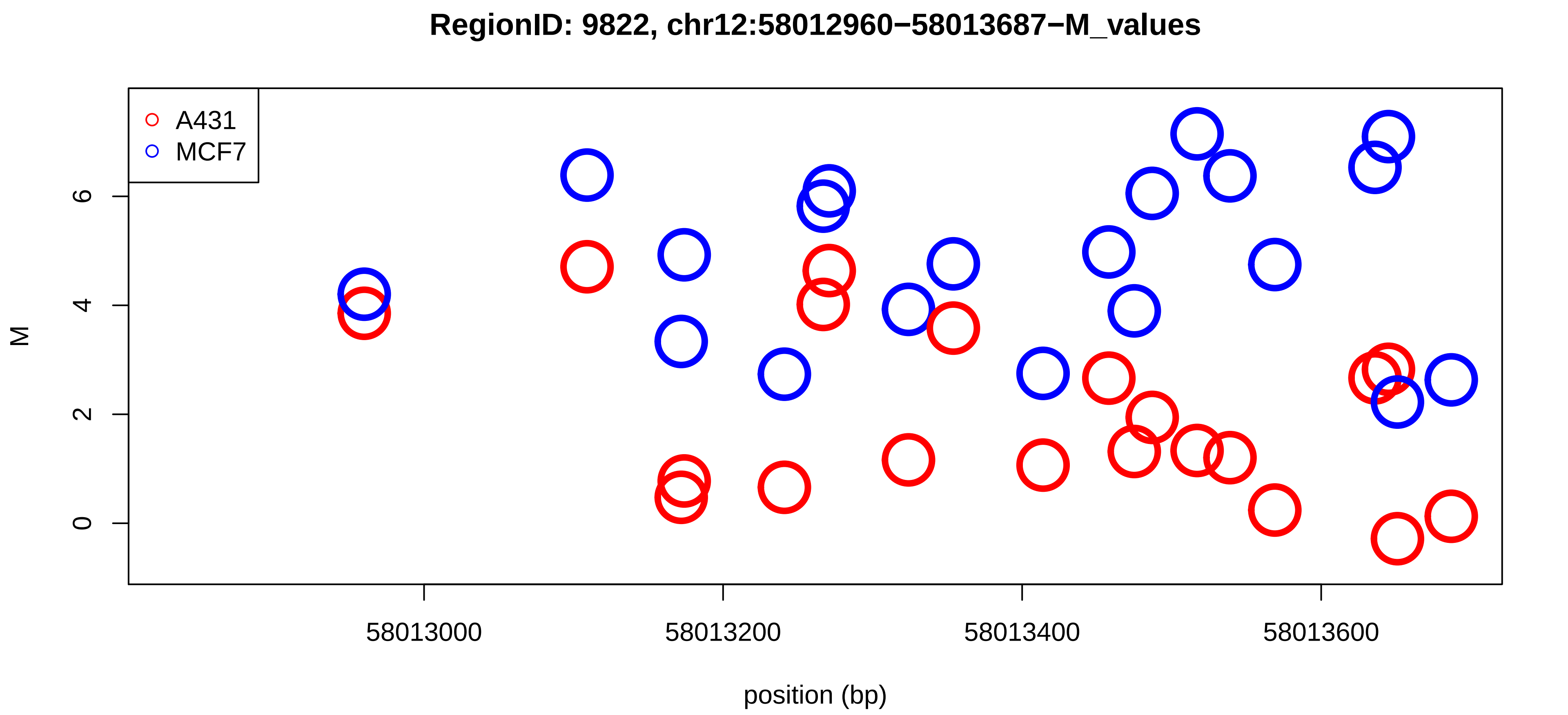
RegionID: 9822, chr12:58012960−58013687−M_values
A431
MCF7
M
0
2
4
6
58013000
58013200
58013400
58013600
position (bp)

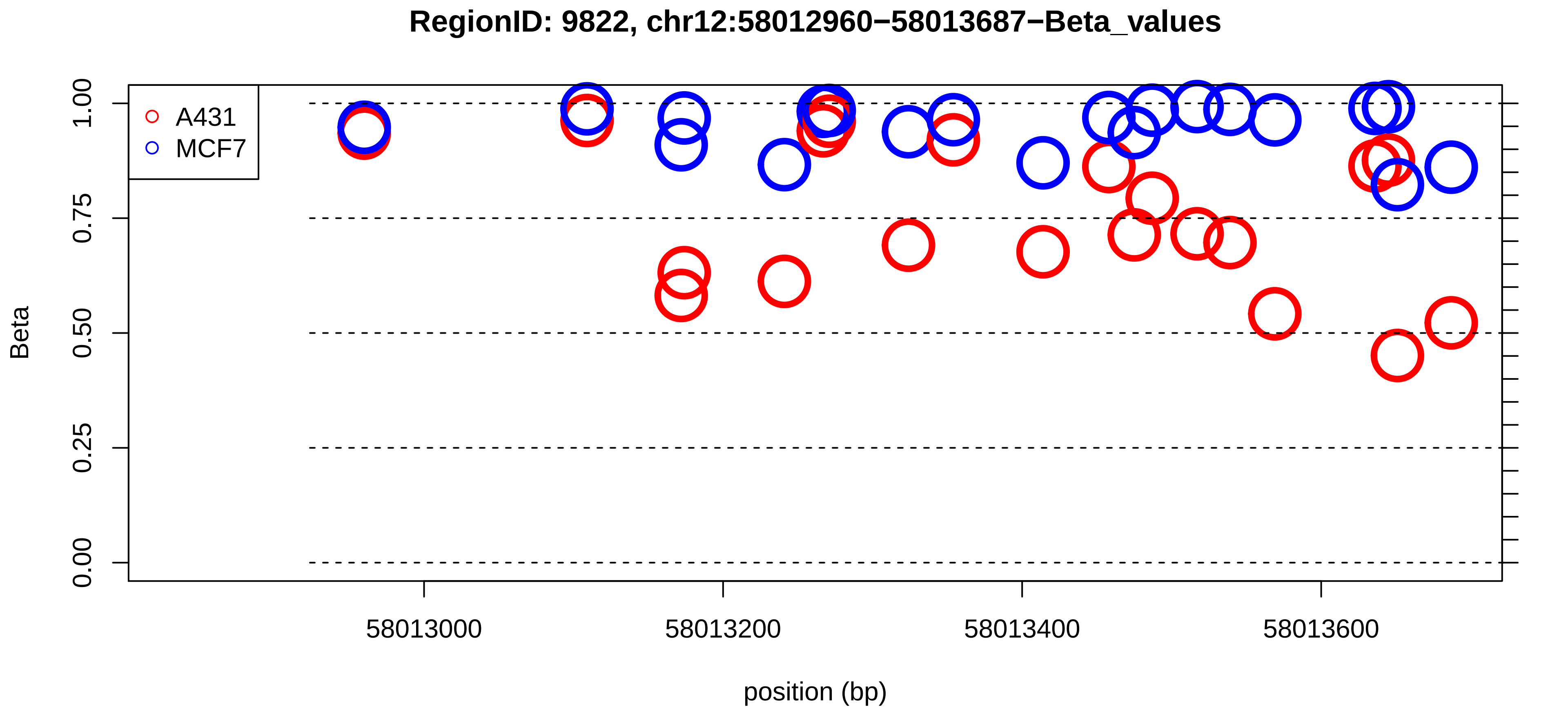
RegionID: 9822, chr12:58012960−58013687−Beta_values
A431
MCF7
Beta
0.00
0.25
0.50
0.75
1.00
58013000
58013200
58013400
58013600
position (bp)

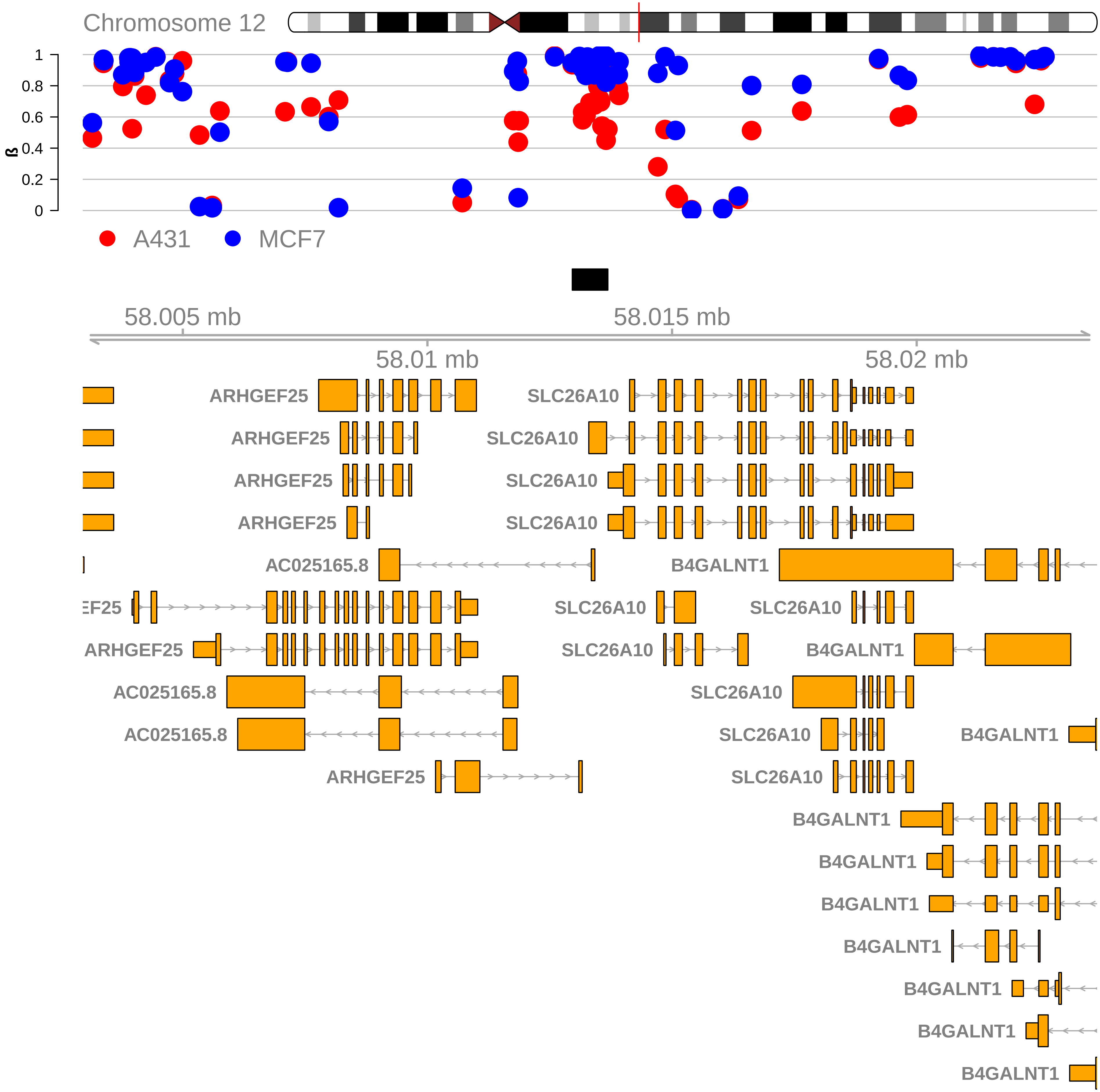
Chromosome 12
1
0.8
0.6
ß 0.4
0.2
0
A431
MCF7
58.005 mb
58.015 mb
58.01 mb
58.02 mb
ARHGEF25
SLC26A10
ARHGEF25
SLC26A10
ARHGEF25
SLC26A10
ARHGEF25
SLC26A10
AC025165.8
B4GALNT1
EF25
SLC26A10
SLC26A10
ARHGEF25
SLC26A10
B4GALNT1
AC025165.8
SLC26A10
AC025165.8
SLC26A10
B4GALNT1
ARHGEF25
SLC26A10
B4GALNT1
B4GALNT1
B4GALNT1
B4GALNT1
B4GALNT1
B4GALNT1
B4GALNT1
